# Nonlinear Dynamics and Chaos of Microcantilever-Based TM-AFMs with Squeeze Film Damping Effects

**DOI:** 10.3390/s90503854

**Published:** 2009-05-20

**Authors:** Wen-Ming Zhang, Guang Meng, Jian-Bin Zhou, Jie-Yu Chen

**Affiliations:** State Key Laboratory of Mechanical System and Vibration, School of Mechanical Engineering, Shanghai Jiao Tong University, 800 Dongchuan Road, Shanghai 200240, China; E-Mails: gmeng@sjtu.edu.cn (G.M.); giantbean@sjtu.edu.cn (J.-B.Z.); jerrysmiling@hotmail.com (J.-Y.C.)

**Keywords:** TM-AFM, microcantilever, squeeze film damping, Lennard-Jones (LJ) potential

## Abstract

In Atomic force microscope (AFM) examination of a vibrating microcantilever, the nonlinear tip-sample interaction would greatly influence the dynamics of the cantilever. In this paper, the nonlinear dynamics and chaos of a tip-sample dynamic system being run in the tapping mode (TM) were investigated by considering the effects of hydrodynamic loading and squeeze film damping. The microcantilever was modeled as a spring-mass-damping system and the interaction between the tip and the sample was described by the Lennard-Jones (LJ) potential. The fundamental frequency and quality factor were calculated from the transient oscillations of the microcantilever vibrating in air. Numerical simulations were carried out to study the coupled nonlinear dynamic system using the bifurcation diagram, Poincaré maps, largest Lyapunov exponent, phase portraits and time histories. Results indicated the occurrence of periodic and chaotic motions and provided a comprehensive understanding of the hydrodynamic loading of microcantilevers. It was demonstrated that the coupled dynamic system will experience complex nonlinear oscillation as the system parameters change and the effect of squeeze film damping is not negligible on the micro-scale.

## 1. Introduction

**Figure 1.**
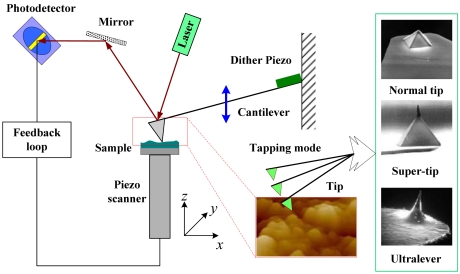
Schematic of the typical TM-AFM setup.

Atomic force microscopy (AFM) has been developed to a nearly ubiquitous tool for studying physics, chemistry, biology, medicine and engineering at the nano-scale [1-5]. AFM could significantly impact many fabrication and manufacturing processes due to its advantages such as 3D topography of nano-fabrication and metrology for MEMS [2]. As a typical dynamic mode, the tapping mode (TM) is widely used in the operation of AFM where the cantilever is driven at a fixed frequency close or equal to the fundamental resonance frequency of vertical bending [4,6]; a schematic of the TM-AFM setup is shown in Figure 1. Moreover, the vibration amplitude of the cantilever is much bigger than the equilibrium separation between the tip and the sample. The TM-AFM has attracted extensive attention due to its ability to deal with compliant materials as well as to overcome adhesion forces.

The inherently and highly nonlinear tip-sample interaction will give rise to complex dynamics of the cantilever in TM-AFM [7]. Nonlinearity is essential in understanding the dynamics of cantilevers as there are many nonlinear forces in TM-AFM, such as the attractive van der Waals forces, the short-range repulsive interactions, contact nonlinearities and capillary forces *et al.* [8-10]. Ashhab *et al.* [11] concluded that chaos in AFM depend on the damping, excitation and tip-sample distance, and suggested that a state feedback control can be used to eliminate the possibility of chaotic behavior. Using nonlinear analysis methods and numerical simulations, Basso *et al.* [12] found that the chaotic behavior may occur via a cascade of period doubling bifurcations. In their studies [11,12], Melnikov theory was used to predict the existence of chaos in AFM. The nonlinear dynamics to frequency sweeps in TM-AFM was simulated using the van der Waals forces and the Derjaguin-Muller-Toporov (DMT) and Johnson-Kendall-Roberts (JKR) contact models [6,13]. Lee *et al.* [6] carried out numerical analysis using modern continuation tools for computational nonlinear dynamics and bifurcation problems where the tip-surface interaction was represented by the van der Waals and DMT contact forces. Nonlinear hysteresis and jumps in the dynamic response were examined as the tip approaches to or retracts from the sample at a fixed excitation frequency [14]. Zitzler *et al.* [15] considered the influence of hysteretic capillary forces in TM-AFM and studied the effect of the relative humidity on the amplitude and phase of the cantilever oscillation. By using the forward-time simulation and numerical continuation techniques [15], Hashemi *et al.* [10] investigated the nonlinear dynamics of a TM-AFM with tip-surface interactions, which include attractive, repulsive, and capillary forces. Hersam [3] conducted experiments to verify the importance of nonlinear dynamics in TM-AFM measurements. Rutzel *et al.* [9] used the Lennard-Jones (LJ) potential to model the tip-surface interactions and carried out a comprehensive investigation to the nonlinear dynamics and stability of the TM-AFM, and the results showed that considering the LJ interaction potential in modeling the dynamics of AFM could improve the qualitative prediction of the real system response.

Nonlinear dynamics and chaos of the cantilever for the TM-AFM remain a challenging and critical issue. After experimental observations, Couturier *et al.* [16] mentioned that the motion of the cantilever could become chaotic under instability conditions. When the cantilever is driven close to the surface of the sample, the squeeze film between the cantilever and the sample surface contributes significantly to the damping and gives rise to the complicated nonlinear behavior [17,18]. When studying the frequency response of AFM cantilevers in liquid media contained in a commercial fluid cell, Motamedi and Wood-Adams [19] found that such systems could exhibit complicated dynamics. However, a rational connection of the tip-sample-interaction and the nonlinear dynamics analysis of the cantilever where the coupled effects of squeeze film damping and hydrodynamic loading are considered has not been presented and addressed satisfactorily. To order to make the TM-AFM achieve good performance, it is necessary to identify and so as to eliminate the possible chaotic motion of the cantilever of the AFM. In this paper, the cantilever is modeled as a single spring-mass-damper system and a nonlinear dynamic model is developed to study the cantilever-sample interaction by using the LJ potential including the long-range attractive forces and short-range repulsive forces. A comprehensive investigation of the nonlinear dynamics and chaos of the TM-AFM is carried out.

The rest of the paper is organized as follows. Section 2 describes the mathematic model of the cantilever vibrating in air considering the effects of hydrodynamic loading and squeeze film damping and the physical model of the cantilever-sample interaction is established in Section 3 using the LJ potential. Numerical results and discussions of the quality factor and resonant frequency of the frequency response, and nonlinear chaos and bifurcation of the dynamic TM-AFM are presented in Section 4. Finally, we end the paper with our conclusions in Section 5.

## 2. Micro-Cantilever Vibrating in Air

### 2.1. Hydrodynamic Loading Effect

The gas flow around the cantilever is assumed to be incompressible and the Navier-Stokes equation is given by [20]:

(1) $$\rho \frac{\partial \text{v}}{\partial t}+\rho \left(\text{v}\cdot \nabla \right)\text{v}=-\nabla p+\mu \nabla^{2}\text{v}$$

where *ρ*, **v**, *p* and *μ* are the density, velocity, pressure and viscosity of the gas, respectively.

It has been found that the wavelength of vibration greatly exceeds the dominant length scale in the flow [20,21]. The nominal width *B* is the dominant length scale in the gas flow and the appropriate Reynolds number *R_e_* can be expressed as [21]:

(2) $$R_{e}=\frac{\rho \omega B^{2}}{4\mu }$$

where *ω* is a characteristic radial vibration frequency and *R_e_* is a normalized Reynolds number which indicates the importance of viscous forces relative to inertial forces in the gas fluid. It can be found that the gas could be considered to be non-viscous in the limit as *R_e_* → ∞, and the non-viscous gas model is applicable for practical cases where *R_e_* ≫ 1 [21]. However, if *ω* is close to the resonant frequency of the cantilever in vacuum, a reduction in dimensions of the beam will result in a reduction in *R_e_*.

The surrounding viscous fluid medium plays an important role on the dynamics of the cantilever in AFM, Sader *et al.* [21,22] studied the effect of viscous fluid medium on the AFM cantilever and found that the shift in resonant frequency of the cantilever from vacuum to fluid (gas or liquid) was strongly dependent on both the density and viscosity of the fluid. For a rectangular cantilever beam [21], when the quality factor of the fundamental mode of the cantilever in gas exceeds 1, which is typically satisfied when the cantilever is placed in air, the relationship between the vacuum resonant frequency *ω_vac_* and the resonant frequency in gas *ω_gas_* can be written as [20]:

(3) $$\frac{\omega_{\text{gas}}}{\omega_{\text{vac}}}={\left(1+\frac{\pi \rho B}{4\rho_{c}H}\Gamma_{r}(\omega_{\text{gas}})\right)}^{-1/2}={\left(1+\frac{\pi }{4}\Pi \Gamma_{r}(\omega_{\text{gas}})\right)}^{-1/2}$$

where *ρ_c_* and *H* are the density and thickness of the cantilever, respectively, and the natural scaling parameter is $\Pi =\frac{\rho B}{\rho_{c}H}$, which is defined as the ratio of the added mass of the gas to the mass of the cantilever.

To study the effect of hydrodynamic loading on the dynamics of the cantilever, the hydrodynamic functions Γ(*ω*), which represents the real and imaginary pressure of the surrounding on the cantilever in two dimensions, is given by [20,21]:

(4) $$\Gamma =\Gamma_{r}+j\Gamma_{i}$$

where $\Gamma_{r}=a_{1}+\frac{a_{2}}{\sqrt{R_{e}}}$ and $\Gamma_{i}=\frac{b_{1}}{\sqrt{R_{e}}}+\frac{b_{2}}{R_{e}}$, and a_1_ = 1.0553, a_2_ = 3.7997, b_1_ = 3.8018, and b_2_ = 2.7364 are selected from [20].

The quality factor for the bending modes can be written as [20]:

(5) $$Q_{\text{gas}}=\frac{\frac{4\mu }{\pi \rho B^{2}}+\Gamma_{r}(\omega_{\text{gas}})}{\Gamma_{i}(\omega_{\text{gas}})}=\frac{\frac{4}{\pi }\cdot \frac{1}{\Pi }+\Gamma_{r}(\omega_{\text{gas}})}{\Gamma_{i}(\omega_{\text{gas}})}$$

For small amplitude of the normal vibration of the cantilever, the local interaction stiffness in the vertical direction *k_n_* and damping coefficient *C_n_* is given by [23]:

(6) $$k_{n}=k_{L}\left(\frac{A_{n0}}{A_{n}}\cos\varphi -1\right)$$

And:

(7) $$C_{n}=-\frac{k_{L}A_{n0}}{A_{n}\omega }\sin\varphi$$

where *A_n_*_0_ is the free amplitude in the normal direction, *A_n_* is the measured amplitude, *φ* is the measured phase of the cantilever, *ω* is the drive frequency, and *k_L_* is the normal bending stiffness and $k_{L}=\frac{EBH^{3}}{4L^{3}}$, in which *E, B, H* and *L* are the elastic modulus, width, thickness and length of the cantilever, respectively. When the cantilever in AFM operates far below the resonance, the phase angle is close to zero, then *k_n_* and *C_n_* become:

(8) $$k_{n}=k_{L}\left(\kappa -1\right),\;C_{n}=0$$

where *κ* =*A*_*n*0_/*A_n_*.

### 2.2. Squeeze Film Damping Effect

When the cantilever is driven close to the sample, the squeeze film between the cantilever and the surface of the sample causes significant damping except for the fluid dissipation at the edges and above the cantilever. For the damping problems encountered at micro-scale [24], the squeeze film damping can be expressed by the nonlinear Reynolds equation:

(9) $$\frac{\partial }{\partial \xi }\left(\rho \frac{h^{3}}{\mu }\frac{\partial p}{\partial \xi }\right)+\frac{\partial }{\partial \zeta }\left(\rho \frac{h^{3}}{\mu }\frac{\partial p}{\partial \zeta }\right)=12\frac{\partial \left(h\rho \right)}{\partial t}$$

Under the basic assumptions including the gas in the gap has been regarded as a continuum and the gas undergoes an isothermal process, the squeeze film damping can be modeled as:

(10) $$\frac{p_{a}h^{2}}{12\mu_{\text{eff}}}\left[\frac{\partial^{2}}{\partial \xi^{2}}\left(\frac{p}{p_{a}}\right)+\frac{\partial^{2}}{\partial \zeta^{2}}\left(\frac{p}{p_{a}}\right)\right]=\frac{\partial }{\partial t}\left(\frac{\xi }{h}\right)+\frac{\partial }{\partial t}\left(\frac{p}{p_{a}}\right)$$

where the pressure *p* is small compared to the ambient pressure *p_a_* (*p_a_*=1.013×10^5^ *pa*), *μ_eff_* is the effective gas viscosity, and *h* is the gap between the cantilever and the surface of the sample. At micro-scale, when the ratio between the mean free path of the air *λ* and the film thickness *h*, i.e., Knudsen number *K_n_* = *λ/h*, is not small, the no-slip boundary condition at the interface may be inadequate. Considering the effect of slip flow, the effective viscosity *μ_eff_* can be expressed as [25]:

(11) $$\mu_{\text{eff}}=\frac{\mu }{1+9.638K_{n}^{1.159}}$$

where *μ* is the viscosity coefficient at 1 atm.

For the normal motion of parallel plates, *h* and *μ_eff_* are not functions of the position. Equation (10) can be simplified as:

(12) $$\frac{h^{3}}{12\mu_{\text{eff}}}\left[\frac{\partial }{\partial \xi }\left(p\frac{\partial p}{\partial \xi }\right)+\frac{\partial }{\partial \zeta }\left(p\frac{\partial p}{\partial \zeta }\right)\right]=\frac{\partial (ph)}{\partial t}$$

Then, the damping pressure can be obtained by direct integration with the boundary conditions, i.e.:

(13) $$p(\xi ,t)=-\frac{6\mu_{\text{eff}}}{h^{3}}\left[{\left(\frac{B}{2}\right)}^{2}-\xi^{2}\right]\cdot \frac{dh}{dt}$$

The damping force on the cantilever is:

(14) $$F_{s}=\int_{-B/2}^{B/2}p(\xi ,t)Ld\xi =-\frac{\mu_{\text{eff}}\;B^{3}L}{h^{3}}\cdot \frac{dh}{dt}$$

and the coefficient of damping force is given by:

(15) $$C_{s}=\frac{\mu_{\text{eff}}\;B^{3}L}{h^{3}}$$

## 3. The Physical Model

**Figure 2.**
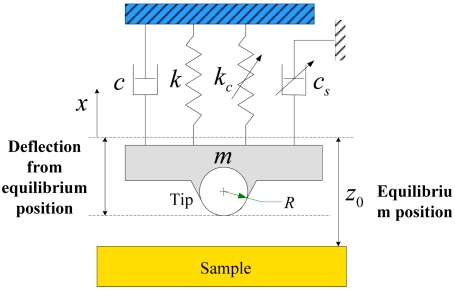
Schematic of the lumped spring-mass-damping model for the TM-AFM cantilever vibrating near a sample surface.

The AFM is composed of an elastic cantilever and the achievable sensitivity and resolution of AFM depend largely on the geometry of the cantilever [11,26]. Considering only the first vibration mode, the cantilever can be modeled as a simplified spring-mass-damping system, as shown in Figure 2. The tip is modeled as a sphere of radius *R*, and the cantilever-sample distance is characterized by *z*_0_, which is the distance between the equilibrium position of the cantilever and the sample when only the gravity acts on it. The cantilever position is given by *x* measured from the equilibrium position.

The LJ potential models the dispersive van der Waals forces as well as the short-range repulsive exchange interactions between two molecules [9]. The interaction between a cantilever tip and sample surface can be modeled as the interaction between a sphere and a flat surface. The tip-sample interaction is modeled by the LJ potential given by [9,11]:

(16) $$U_{LJ}\;(x,z_{0})=\frac{A_{1}R}{1260{\left(z_{0}+x\right)}^{7}}-\frac{A_{2}R}{6\left(z_{0}+x\right)}$$

where *A*_1_ and *A*_2_ are the Hamaker constants for the attractive and repulsive potentials, respectively. The Hamaker constants are defined as *A*_1_ = π^2^*ρ*_1_*ρ*_2_*c*_1_ and *A*_2_ = π^2^*ρ*_1_*ρ*_2_*c*_2_, in which *ρ*_1_ and *ρ*_2_ are the densities of the two interaction components, and *c*_1_ and *c*_2_ are the interaction constants, respectively.

When the cantilever is driven close to the sample, there are three different kinds of forces including the spring force, the van deer Waals attractive force which is proportional to the inverse square power of the distance between the cantilever tip and the sample, and the repulsive force which is proportional to the inverse eighth power of the distance between the tip and the sample. The LJ force can be defined as the sum of the attractive and repulsive forces and is expressed as [9]:

(17) $$F_{LJ}=-\frac{\partial U_{LJ}\;(x,z_{0})}{\partial (x+z_{0})}=\frac{A_{1}R}{180{\left(z_{0}+x\right)}^{8}}-\frac{A_{2}R}{6{\left(z_{0}+x\right)}^{2}}$$

During the AFM operates in the TM, a low-dimensional model reduction can provide an accurate description of the cantilever dynamics. As shown in Figure 2, the cantilever is driven by the harmonic driving force, the tip-sample interaction force *F_LJ_* (LJ force) and the force due to squeeze film damping *F_s_*. The governing equation of the motion of the cantilever can be determined by:

(18) $$m\ddot{x}+c\dot{x}+kx+k_{c}x^{3}=F_{LJ}\;(x,z_{0})+F_{s}\;\left(x,\dot{x},z_{0}\right)+f_{0}\cos\omega t$$

where *x* is the instantaneous displacement of the cantilever tip measured from the equilibrium tip position in the absence of external forces with positive values toward the sample surface, and *ẋ* and *ẍ* are the instantaneous velocity and acceleration of the cantilever tip,. *m, k* and *c* are the equivalent mass, spring stiffness and damping coefficients of the cantilever in air, respectively, and *k* = *k_L_* + *k_n_* and *c* = *mω*_1_/*Q* + C*_n_*, in which $\omega_{1}=\sqrt{k_{L}/m}$ is the first-order mode frequency, and *k_c_* is the nonlinear cubic stiffness. *f*_0_ and *ω* are the amplitude and angular frequency of the harmonic driving force.

The damping force *F_s_*(*x, ẋ*, *z*_0_) due to the squeeze film on the cantilever can be obtained from equation (14) and is given by:

(19) $$F_{s}\;\left(x,\dot{x},z_{0}\right)=c_{s}\dot{x}=\frac{\mu_{\text{eff}}\;B^{3}L}{{\left(x+z_{0}\right)}^{3}}\dot{x}$$

In order to facilitate the investigation of the qualitative behaviors of the dynamic system, the equilibrium distance variable *Z_s_* is defined and *Z_s_* = (3/2)(2*D*)^1/3^ [11], in which *D* = *A*_2_ *R*/(6*k*).

Introducing dimensionless variables:

(20) $$\tau =\omega_{1}t,\;y=\frac{x}{Z_{s}},\;\dot{y}=\frac{\dot{x}}{\omega_{1}Z_{s}},\;d=\frac{4}{27},\;\alpha =\frac{z_{0}}{Z_{s}},\;\beta =\frac{k_{c}}{k}Z_{s}^{2},\;\gamma =\frac{c_{s}}{m\omega_{1}},\;\chi =\frac{\mu_{\text{eff}}\;B^{3}L}{m\omega_{1}Z_{s}^{3}},\;F_{0}=\frac{f_{0}}{kZ_{s}},\;\Omega =\frac{\omega }{\omega_{1}},\;\sigma ={\left(\frac{A_{1}}{A_{2}}\right)}^{1/6},\;\sum =\frac{\sigma }{Z_{s}},\;\zeta =\frac{1}{Q}$$

Then, the LJ force *F_LJ_* and the squeeze film damping force *F_s_* can be rewritten as:

(21) $$F_{LJ}=-\frac{d}{{\left(\alpha +y\right)}^{2}}+\frac{\sum^{6}d}{30{\left(\alpha +y\right)}^{8}}$$

And:

(22) $$F_{s}=\chi \cdot \frac{1}{{\left(\alpha +y\right)}^{3}}\dot{y}$$

Therefore, the dynamic equation of the system can be given by:

(23) $$\ddot{y}+\zeta \dot{y}+y+\beta y^{3}=-\frac{d}{{\left(\alpha +y\right)}^{2}}+\frac{\sum^{6}d}{30{\left(\alpha +y\right)}^{8}}+\varepsilon \left(\Gamma \cos\Omega \tau -\eta \frac{1}{{\left(\alpha +y\right)}^{3}}\dot{y}\right)$$

where, Γ = *F*_0_/*ε*, Δ = *γ/ε, η* = *χ/ε*, and *ε* is a small perturbation.

## 4. Results and Discussion

This section aims at numerically investigating the characteristics and nonlinear dynamics of a TM-AFM cantilever-sample system driven by the harmonic excitation. The general properties of the cantilever and interaction properties with the respective sample are referred to [9], are listed in Table 1. The 4th order Runge-Kutta method is used to integrate the set of Equation (23). A small integration step (2π/200) has to be chosen to ensure a stable solution and to avoid the numerical divergence at the points where derivatives of *F_LJ_* and *F_s_* are discontinuous. The effects of system parameters on the dynamic behavior of the cantilever vibrating system are investigated by using the bifurcation diagram, Poincaré maps, largest Lyapunov exponent, phase portraits, time histories and amplitude spectrum.

### 4.1. Effect of External Forcing Term Γ

**Figure 3.**
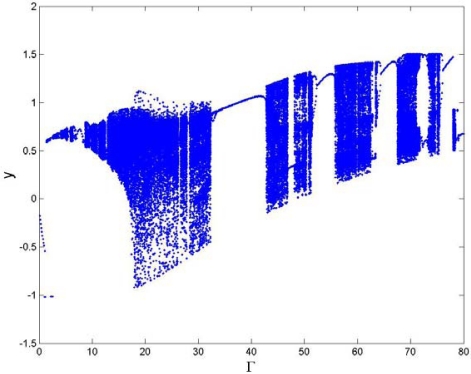
Bifurcation diagram of the amplitude of the external forcing term Γ.

**Figure 4.**
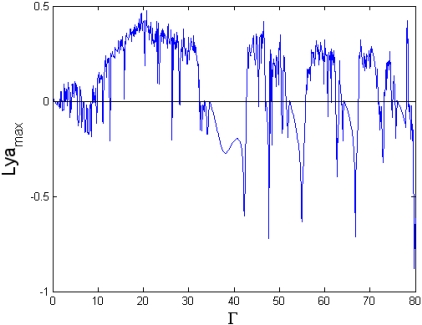
Largest Lyapunov exponent map of the amplitude of the external forcing term Γ.

The external forcing term is one of the most important parameters affecting the dynamic characteristics of the TM-AFM tip-sample system. Figure 3 and Figure 4 show the bifurcation diagram and largest Lyapunov exponent map of the dynamic system where the amplitude of the external excitation is the control parameter and the small perturbation *ε* = 0.1 is added.

**Figure 5.**
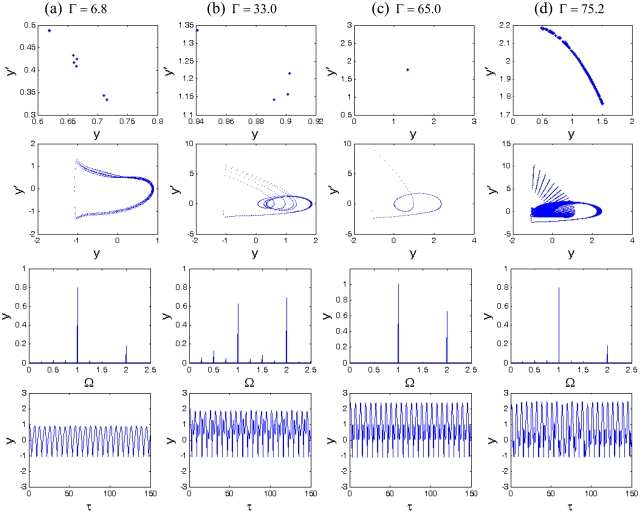
The Poincaré maps, phase portraits, amplitude spectrums and time histories of different Γ.

It can be seen from Figure 3 that the system responses contain periodic and chaotic motions alternately at the interval of 0 < Γ< 80. When Γ = 6.8, the vibration amplitude of the cantilever is small, the motion is synchronous with period-eight (P-8), and eight points are correspondingly displayed in the Poincaré map, as shown in Figure 5(a). With the increase of the amplitude of the forcing term Γ, the motion becomes synchronous with period-two (P-2) at Γ = 33.0, as illustrated in Figure 5(b). Moreover, the period-1 motion with only one isolated point in Poincaré map and one circle in phase portrait can be observed at Γ = 65.0 in Figure 5(c), and the corresponding largest Lyapunov exponent becomes negative, according to Figure 4. At Γ = 75.2, the chaos is shown in Figure 5(d), the strange attractor has a fractal structure in Poincaré map, and it can be found in Figure 4 that the corresponding largest Lyapunov exponent is positive. Therefore, as the amplitude of the forcing term increases, the changes of the system responses are very complex, with alternative periodic and chaotic motions.

### 4.2. Effect of Squeeze Film Damping η

**Figure 6.**
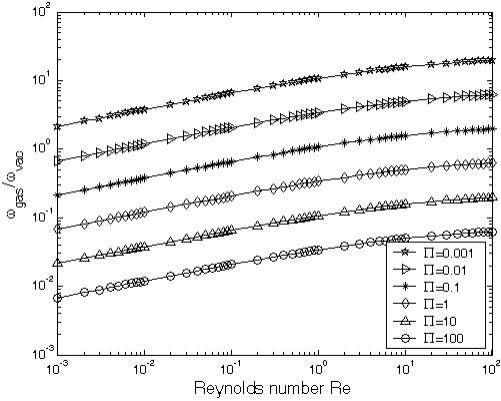
The relationship between the vacuum resonant frequency *ω_vac_* and the resonant frequency in gas *ω_gas_* (*ω_gas_/ω_vac_*) as a function of the Reynolds number *R_e_* at different natural scaling parameter ∏.

**Figure 7.**
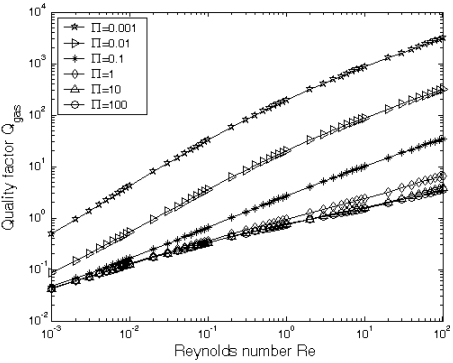
The quality factor *Q_gas_* as a function of the Reynolds number *R_e_* for the fundamental mode at different natural scaling parameter ∏.

At the micro-scale, the squeeze film damping coefficient and ratio are the key parameters for the dynamic responses of the micro-devices. The larger the squeeze film damping is, the higher the noise level results. The ratio of the fundamental resonant frequency in gas to that in vacuum *ω_gas_/ω_vac_* is numerically calculated from Equation (3). Figure 6 gives the ratio of resonant frequencies in vacuum and gas *ω_gas_/ω_vac_* as a function of the Reynolds number *R_e_* at different natural scaling parameter. ∏ It could be found that *ω_gas_/ω_vac_* is increasing with the increase of Reynolds number *R_e_* and *ω_gas_/ω_vac_* decreases with the increase of the scaling parameter ∏. Figure 7 gives the quality factor *Q_gas_* as a function of both natural scaling parameters, *R_e_* and ∏, which can be obtained directly from Equation (5). It is indicated that the quality factor *Q_gas_* increases with the increase of the Reynolds number *R_e_* and the decrease of the natural scaling parameter ∏. However, when the natural scaling parameter ∏ tends to be a larger value, i. e., ∏ = 10, the quality factor *Q_gas_* has small change. For example, when ∏ changes from 10 to 100, the quality factor *Q_gas_* has very small change, as shown in Figure 7.

**Figure 8.**
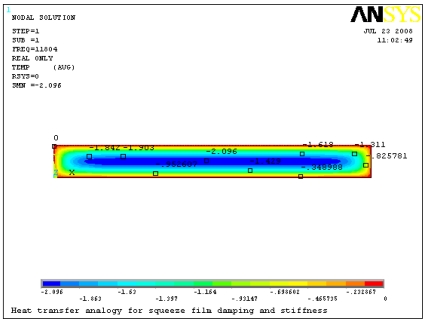
Pressure distributions of the cantilever at the resonant frequency.

Microstructures undergoing motion transverse to a fixed plate exhibit damping effects that should be considered in the dynamics simulation. The heat transfer analogy is applied to obtain the damping and stiffness coefficients of the microstructures with PLANE 55 thermal elements in ANSYS 11.0 [28]. The effective damping and stiffness coefficients are determined by the finite element thermal analogy approach and theoretic analyses at different operation frequencies and the results are listed in Table 2. It can be seen that the damping coefficient decreases tardily with the increase of the operation frequency and the stiffness coefficient increases fleetly at the same time with slip and without slip. Meanwhile, the damping and stiffness coefficients with slip effect are smaller than those without slip. Figure 8 shows the pressure distribution of the damping component with slip at the resonant frequency. The pressure distribution is approximately parabola in the directions of length and width, and the peak appears at the center of the film.

**Figure 9.**
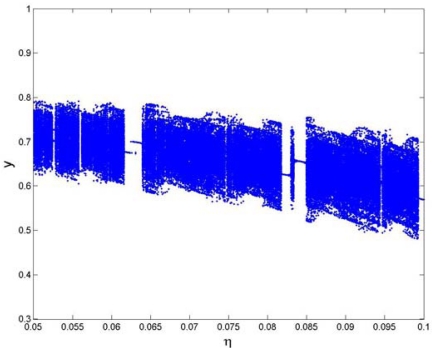
Bifurcation diagram of the squeeze film damping ratio *η*.

**Figure 10.**
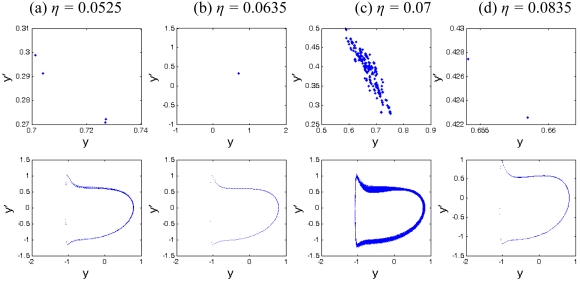
The Poincaré maps and phase portraits of different squeeze film damping ratios *η*.

Figure 9 displays the bifurcation diagrams of squeeze film damping ratio *η* in the range of 0.05 < *η* < 0.1 for the coupling nonlinear dynamic system, from which it can be that the system response changes between periodic and chaotic motions alternately. Figure 10 shows the Poincaré maps and phase plane portraits of different squeeze film damping ratio *η* on the responses of the coupling system. The system response starts synchronous motion with period-4 at *η* = 0.0525 (shown in Figure 10a), and then becomes synchronous motion with period-1 at *η* = 0.0635(shown in Figure 10b), and then leaves synchronous motion with period-1 and enters chaotic motion at *η* = 0.07, which can be seen in Figure 10c. The strange attractor has a fractal structure and the corresponding largest Lyapunov exponent is positive. As indicated in Figure 10d, with the increase of squeeze film damping ratio, the system response becomes synchronous motion with period-1 from chaotic motion. Therefore, the effect of squeeze film damping on the system response cannot be neglected for structures at the micro-scale.

### 4.3. Effect of material property parameter Σ

**Figure 11.**
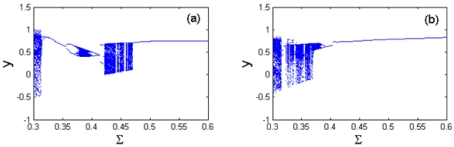
Bifurcation diagram of th 1 cm e material property parameter *Σ* at different cubic stiffness ratios: (a) *β* = 0.3; (b) *β* = 0.5.

At micro-scale, the material properties of the AFM tip and sample play an important role on the surface force between them and, as a result, the dynamic response of the tip-sample system displays very rich nonlinear characteristics. Figure 11 is the bifurcation diagram of the material property parameter *Σ* for the coupling nonlinear dynamic system with different cubic stiffness ratios and squeeze film damping ratios. The system parameters are taken as follows: equilibrium parameter *α* = 1.2, excitation frequency ratio Ω = 1, integration step numbers *N* = 200 and the bifurcation step ΔΣ = 0.005.

Figure 11a shows the bifurcation diagram of the material property parameter in the range of 0.3 < *Σ* < 0.42 with the cubic stiffness ratio *β* = 0.3. The response of the coupled nonlinear system undergoes a complete process from chaotic motion through period-2, period-1, period-4 and period-8 motions to steady-state motion with period-1 by the forms of period-doubling and anti period-doubling bifurcations. At the interval of 0.42 < *Σ* < 0.47, the system response alters between chaotic motion with long time and periodic motion with short time. When *Σ* increases to *Σ* > 0.47, the system response becomes synchronous motion with period-1. With the change of the cubic stiffness ratio of the cantilever tip from *β* = 0.3 to *β* = 0.5, the response of the coupled system undergoes the process of chaotic and periodic motions alternatively. It comes into period-2 motion from chaotic motion with anti period-doubling bifurcation, and then enters period-1 motion in a large range of material property parameter (*Σ* > 0.39), as illustrated in Figure 11b. In addition, it is found that the chaotic motion disappears at higher *Σ* with the increase of cubic stiffness ratio (from *β* = 0.3 to *β* = 0.5). Therefore, with the changes of the measured samples in experimental tests, the values of *Σ* vary accordingly, and as a result the response of the coupled system displays various nonlinear dynamic behaviors.

**Figure 12.**
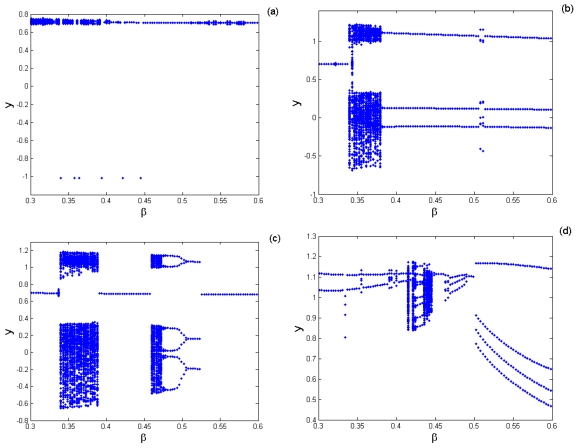
Bifurcation diagram of the cubic stiffness ratio *β* at different combinations of squeeze film damping ratios, material parameters and equilibrium parameters: (a) *η* = 0.08, Σ = 0.3, *α* = 1.2; (b) *η* = 0.08, Σ = 0.5, *α* = 1.2; (c) *η* = 0.14, Σ = 0.5, *α* = 1.2; (d) *η* = 0.14, Σ = 0.5, *α* = 1.6.

Figure 12 is the bifurcation diagram of the cubic stiffness ratio *β* on the response of TM-AFM tip-sample system at the interval of 0.3 < *β* < 0.6 for various combinations of squeeze film damping ratios, material and equilibrium parameters, and the bifurcation step Δ*β* = 0.001. It can be observed from Figure 12(a) that the response of the coupled system has a complete process from chaotic motion through periodic motion and chaotic motion to period-1 motion. At the interval of 0.3 < *β* < 0.43, the system response enters periodic motion from chaotic motion, then it becomes chaotic motion again, and finally it comes into steady-state motion with period-1 in the range of 0.43 < *β* < 0.6. With the increase of squeeze film damping *η* (*η* = 0.14), the system response changes noticeably and it mainly contains the periodic components, such as period-1, period-3 and period-6 motions, as illustrated in Figure 12(c). As the equilibrium parameter *α* increases, the chaotic components of the system response decrease, while the periodic components increase and contain period-2, period-4 and period-8 motions with the case of *α* = 1.6, as shown in Figure 12(d).

**Figure 13.**
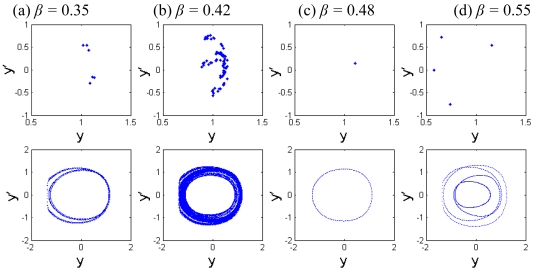
The Poincaré maps and phase portraits of different cubic stiffness ratios *β*.

The Poincaré maps and phase portraits for different cubic stiffness ratios are displayed in Figure 13. When the cubic stiffness ratio is small, i.e., *β* = 0.35, the exhibited motion is period-6 motion, six points and circles can be seen in the Poincaré map and phase portrait respectively from Figure 13(a). As the cubic stiffness ratio becomes larger, the closed circle is decomposed and the points in the Poincaré map gradually scatter. At *β* = 0.42, the system response comes into chaotic motion, and then the points of the attractor are decomposed again and finally converge to one point. When *β* = 0.48, a synchronous motion with period-1 can be observed. Then the system response comes into period-4 motion. It is indicated that the components of chaotic motions become wider with the increase of the squeeze film damping. Material properties of the tip and sample and equilibrium coefficient ratio play very important role in the nonlinear dynamics of the coupled system.

### 4.4. Effect of Equilibrium Parameter α

**Figure 14.**
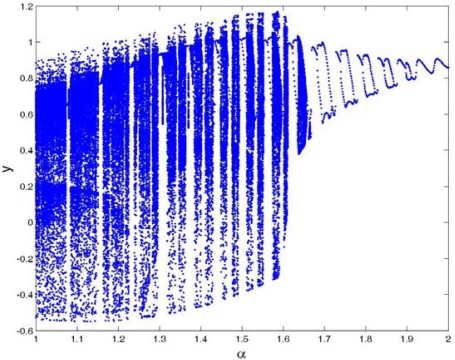
Bifurcation diagram of the equilibrium parameter *α*.

**Figure 15.**
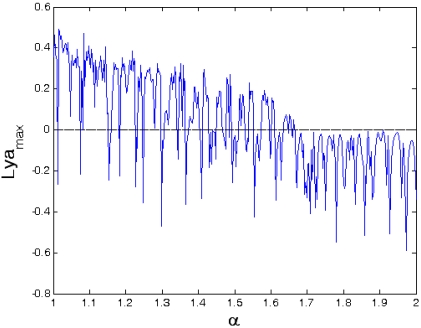
Largest Lyapunov exponent map of the equilibrium parameter *α*.

Equilibrium coefficient ratio *α* is one of the important parameters for determining the equilibrium position of the cantilever tip in AFM and it becomes the key factor to reflect the dynamic responses of the tip-sample model. The dynamic behavior of the coupled system depends on the value of *α*. Figures 14 and 15 give the bifurcation diagram and largest Lyapunov exponent map of the dynamic system with the control parameter of the equilibrium parameter *α* at Ω = 1. It can be seen from Figure 14 that the dynamic responses are very complicated, and the components contain periodic and chaotic motions at the interval of 1< *α* < 2. The corresponding largest Lyapunov exponents are alternately positive and negative, as shown in Figure 15.

**Figure 16.**
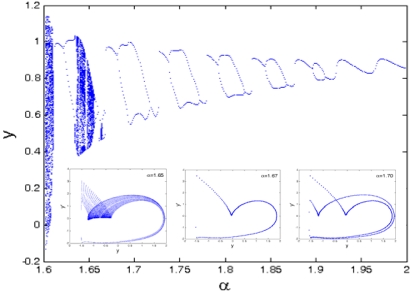
Local bifurcation and Poincaré maps of equilibrium parameter *α*.

To explain the dynamic responses of the system clearly, Figure 16 shows the local bifurcation diagram and Poincaré maps of the dynamic system at the interval of 1.6 < *α* < 2. It can be found that the system responses exhibit the alternation of periodic and chaotic motions. The system response comes into steady-state synchronous motion with period-1 from chaotic motion, and enters period-2 motion from period-1 motion as the equilibrium parameter *α* increases, and then becomes chaotic motion with period-doubling bifurcation. Moreover, at 1.7 < *α* < 1.9, the system response changes between period-1 and period-2 motions alternately. When *α* > 1.9, the system response comes into steady-state synchronous motion with period-1. These phenomena indicate that the dynamic responses of the coupled system are very complex. The cantilever tip can undergo a period-doubling cascade to possible chaos about the original equilibrium. It is demonstrated that, away from the surface, the net force on the tip is always in the downward direction and causes the tip to accelerate the sample until it passes the key point, where the repulsive force plus the spring force becomes larger than the van der Waals force, and then the tip is forced away from the sample.

**Figure 17.**
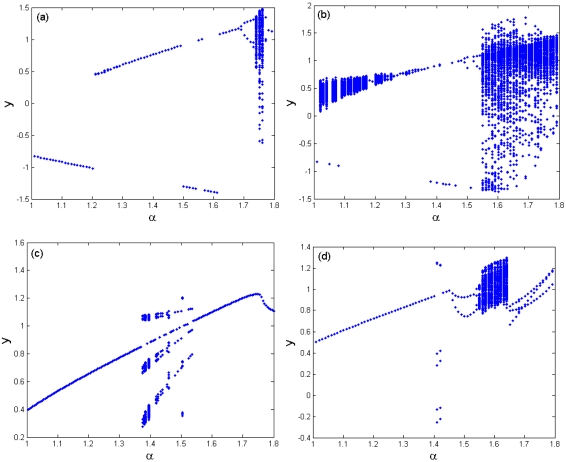
Bifurcation diagram of the equilibrium parameter *α* at different combined material parameters and external forcing terms: (a) Σ = 0.3, Γ = 2; (b) Σ = 0.3, Γ = 4; (c) Σ = 0.5, Γ = 2; (d) Σ = 0.5, Γ = 4.

Figure 17 shows the bifurcation diagram of the equilibrium parameter *α* on the response of TM-AFM tip-sample system at the interval of 1.0 < *α* < 1.8, the squeeze film damping ratio is taken as *η* = 0.008 and the bifurcation step is Δ*α* = 0.001. It can be seen from Figure 17(a) that the response of the coupled dynamic system comes into period-2 motion from period-1 motion and then enters chaotic motion with period-doubling bifurcation. With the increase of *α*, the system responses enters period-2 motion from chaotic motion, and it subsequently becomes period-1 motion again when Σ = 0.3 and Γ = 2. As illustrated in Figure 17(b), the response of the coupled system has a complete process from chaotic motion through periodic motion to chaotic motion with the forms of period-doubling bifurcation and anti period-doubling bifurcations at the interval of < *α* < 1.8 when Σ = 0.3 and Γ = 4. With the increases of the material parameter coefficients, as shown in Figure 17(c) and (d), the system response changes noticeably and it mainly contains the periodic components, such as period-1, period-2, period-3 and period-6 motions. Meanwhile, the chaotic components of the system response decrease and, on the contrary, the periodic components increase. In addition, the chaotic components of the system response shift to the smaller equilibrium parameter. It demonstrates that the components of chaotic motions become wider as the amplitude of external forcing term increases, and the material properties of the tip and sample and equilibrium coefficient ratio affect the nonlinear dynamics of the coupled system.

**Figure 18.**
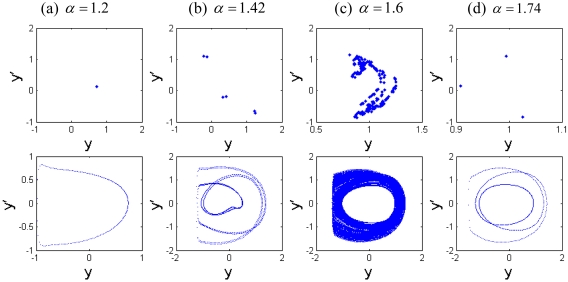
The Poincaré maps and phase portraits of different equilibrium parameters α.

To illustrate the various motions, Figure 18 shows the nonlinear characteristics of the coupled system with the plots of the Poincaré maps and phase portraits for different equilibrium parameters *α* at different conditions. The motion of the coupled system changes between periodic and chaotic motions alternately. At *α* = 1.2, the motion with period-1 represented by a point in the Poincaré maps and characterized by a close curve in phase portraits is shown in Figure 18(a). As illustrated in Figure 18(b), the system response comes into period-6 motion at *α* = 1.42 from synchronous motion with period-1 at *α* = 1.2, as displayed in Figure 18 (a), then leaves period-6 motion and enters chaotic motion at *α* = 1.6, which can be seen from Figure 18(c). The strange attractor has a fractal structure and the corresponding largest Lyapunov exponent is positive. With the increase of the equilibrium parameter coefficient, as shown in Figure 18(d), the system response becomes periodic motion from chaotic motion again, and one can find the period-3 motion marked by three isolated points in Poincaré map and three circles in phase portrait at *α* = 1.74. It is indicated that the components of chaotic motions of the coupled system increases obviously with the increase of the amplitude of the force term. In general, the effect of equilibrium parameter on the system response should be considered for the design of the TM-AFM.

## 5. Conclusions

The cantilever in tapping mode Atomic force microscope (TM-AFM) is one of crucial components and so it is very important to carry out a thorough dynamic analysis for the cantilever to further enhance the performance of the TM-AFM. In this paper, the cantilever-sample interaction and a harmonically forcing term in the TM-AFM have been considered. Numerical simulations have been used to investigate the nonlinear behaviors between the tip and the sample. The chaotic behavior appears to be generated via various system parameters including the amplitude of the external forcing term, equilibrium parameter, squeeze film damping and material property. The dynamic system responses display very rich nonlinear dynamic characteristics under the effects of these parameters and show an alternate changing process among periodic motion, quasi-periodic motion and chaotic motion. The component of the chaotic motion in the system response increases with the increase of the amplitude of the external excitation, but will be weaken by the squeeze film damping. In addition, the increase of the equilibrium parameter coefficient would strengthen the component of the chaotic motion but weaken the component of the periodic motion increases in the system response. It is indicated that the external excitation and squeeze film damping are important for the design of dynamic TM-AFM. It is of significance to understand the cantilever dynamics in air/liquid and promote the development of the next generation of AFMs.

## Figures and Tables

**Table 1.** Parameters of the silicon tip tapping and silicon sample used in the numerical simulations.

**Description**	**Value**
Length	449 μm
Width	46 μm
Thickness	1.7 μm
Tip radius	150 nm
Material density	2,330 kg/m^3^
Young's modulus	176 GPa
Bending stiffness	0.11 N·m^-1^
First-order resonant frequency	11.804 kHz
Quality factor	100
Hamaker constant (Repulsive)	1.3596 × 10^-70^ J·m^6^
Hamaker constant (Attractive)	1.865 × 10^-19^ J

**Table 2.** Effective damping and stiffness coefficients of the squeeze film at different frequencies.

**Frequency *f* (Hz)**	**Damping coefficient**			**Stiffness coefficient**		

	PLANE55		Analytic(slip) [27]	PLANE55		Analytic(slip) [27]
	
	No slip	Slip		No slip	Slip	
1	1.5199e – 4	1.3499e – 4	1.2529e – 4	9.2051e – 10	7.2610e – 10	6.2304e – 10
1 000	1.5199e – 4	1.3499e – 4	1.2529e – 4	9.2051e – 4	7.2610e – 4	6.2304e – 4
11 804	1.5197e – 4	1.3497e – 4	1.2528e – 4	0.1282	0.1012	0.0868
50 000	1.5163e – 4	1.3473e – 4	1.2509e – 4	2.296	1.8118	1.555
100 000	1.5056e – 4	1.3398e – 4	1.2449e – 4	9.117	7.206	6.190
